# SUBJECTIVE HEARING IMPAIRMENT AND LONELINESS IN COMMUNITY-DWELLING OLDER ADULTS

**DOI:** 10.1093/geroni/igz038.1955

**Published:** 2019-11-08

**Authors:** Alison Huang, Jennifer A Deal, George W Rebok, Jay Pinto, Linda J Waite, Frank Lin

**Affiliations:** 1 Department of Mental Health, Johns Hopkins Bloomberg School of Public Health, Baltimore, Maryland, United States; 2 Johns Hopkins University Bloomberg School of Public Health, Baltimore, Maryland, United States; 3 Johns Hopkins Bloomberg School of Public Health, Baltimore, Maryland, United States; 4 The University of Chicago, Chicago, Illinois, United States; 5 Johns Hopkins University, Baltimore, Maryland, United States

## Abstract

Loneliness in older adults is most often attributed to marital and living status, social life factors, and physical health. Hearing impairment, however, is an understudied, potentially modifiable risk factor for loneliness. Older adults with hearing impairment experience difficulties with communication and social functioning, which also could contribute to loneliness. For this analysis, we used data from Wave 2 of the National Social Life, Health, and Aging Project. Participants (N=3,174) were a nationally representative sample of community dwelling older adults aged 62 - 91 years. Poisson regression models with robust variance were used to model the cross-sectional relationship between self-reported hearing impairment and loneliness. We found a dose-response relationship such that individuals reporting very good/good and fair/poor hearing had a 9% [95% CI: 0.93 - 1.28] and 26% [95% CI: 1.10-1.46], respectively, higher prevalence of loneliness compared to individuals reporting excellent hearing, adjusting for chronic conditions, functional and cognitive ability, and demographic factors. Results were robust to exclusion of participants who reported hearing aid use. These findings suggest that self-reported hearing impairment is a strong factor associated with loneliness in older adults. Given the negative implications of loneliness on multiple facets of mental and physical health, functional ability, and premature mortality, efforts to further explore hearing impairment as a causal and modifiable risk factor for loneliness should be undertaken.

